# Supplementary material for: Testis-specific serine/threonine kinase dTSSK2 regulates sperm motility and male fertility in Drosophila
Source: Commun Biol. 2025 May 8;8:710. doi: 10.1038/s42003-025-08163-z (PMC12059139; doi:10.1038/s42003-025-08163-z)
Supplement: Supplementary file 1 — Supplementary information [file 42003_2025_8163_MOESM1_ESM.pdf]

Supplementary information

Testis-Specific Serine/Threonine Kinase dTSSK2 Regulates Sperm Motility and Male Fertility in *Drosophila*

Ju Peng[1], Angyang Sun[1], Jie Zheng[1], Na Zhang[1], Xuedi Zhang[2*], Guanjun Gao[1*]

Author affiliations

[1] School of Life Science and Technology, ShanghaiTech University, Shanghai 201210, PR China
[2] School of Basic Medical Sciences, Suzhou Medical College, Soochow University, Suzhou 215123 Jiangsu Province, China

* Corresponding author

Address correspondence to:
Xuedi Zhang
Department of Cell Biology, School of Basic Medical Sciences, Suzhou Medical College of Soochow University
Suzhou, Jiangsu Province, China 215123
Phone: 86-021-20684529
Fax: 86-021-20684529
E-mail: xdzhang11@suda.edu.cn

Guanjun Gao
School of Life Science and Technology, ShanghaiTech University
393 Middle Huaxia Road
Pudong, Shanghai, China 201210
Phone: 86-021-20685414
Fax: 86-021-20685414
mail: gaogj@shanghaitech.edu.cn

Supplementary Figure S1

a

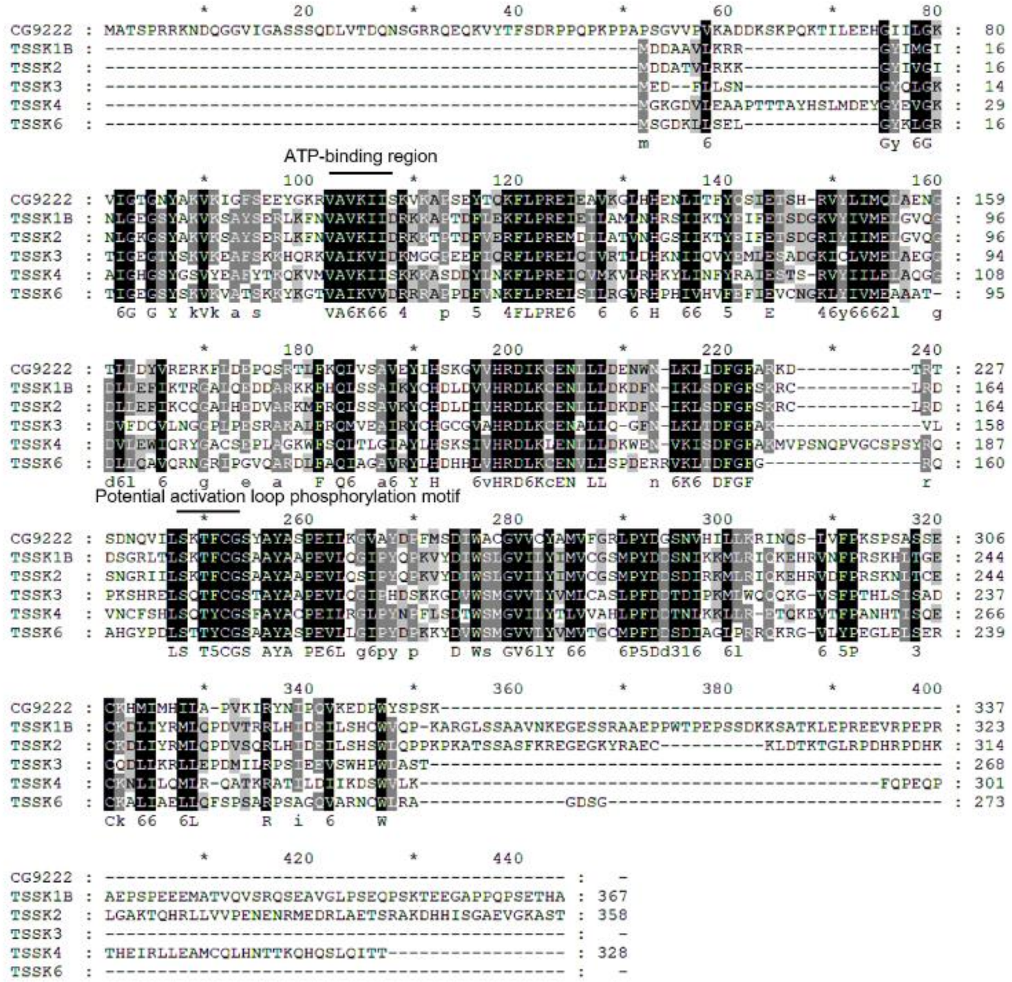

b

	Identity	Similarity
TSSK1B	29%	42%
TSSK2	29%	44%
TSSK3	33%	52%
TSSK4	32%	48%
TSSK6	32%	49%

c

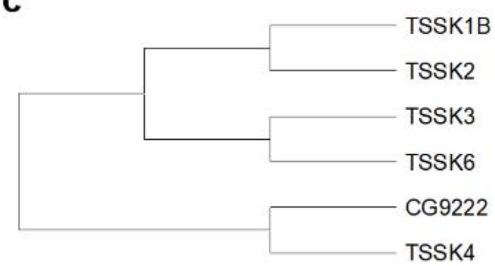

Supplementary Fig. S1. *Drosophila* CG9222 encodes an ortholog of human TSSKs

a Multiple sequence alignment of the protein sequence of *Drosophila* dTSSK2 with those of five human TSSKs (TSSK1B, TSSK2, TSSK3, TSSK4, and TSSK6) using ClustalW. Conserved regions such as the ATP-binding region and potential activation loop phosphorylation motif are indicated. **b** Protein identities and similarities between *Drosophila* dTSSK2 and five human TSSKs analyzed by Blastp. **c** Phylogenetic analysis of *Drosophila* dTSSK2 and five human TSSKs using Phylogeny.

Supplementary Figure S2

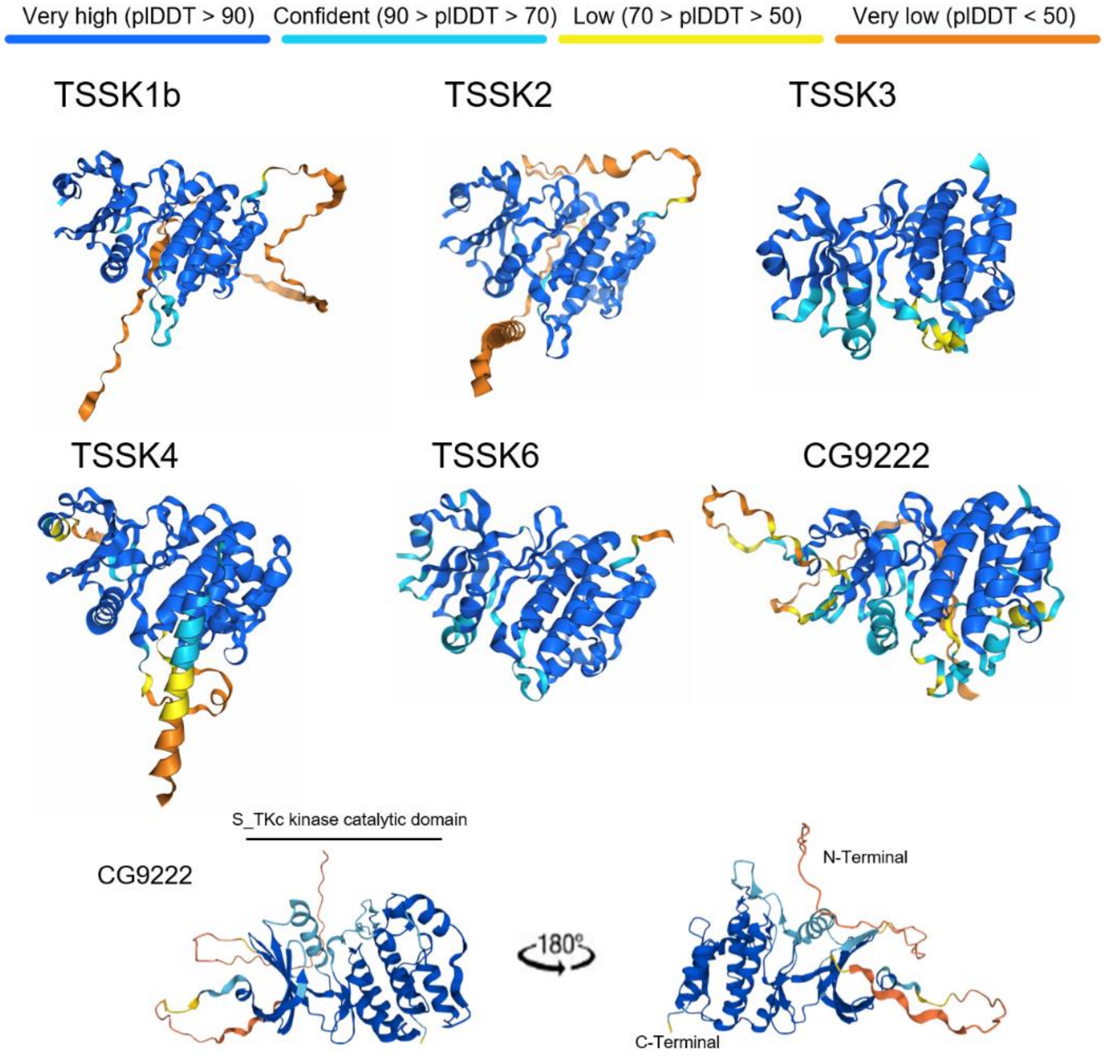

Supplementary Fig. S2. *Drosophila* dTSSK2 encodes an ortholog of human TSSKs Protein structural differences between dTSSK2 and five TSSKs as well as CG14305 predicted in the AlphaFold Protein Structure Database showing conservation of the S_TKc kinase catalytic domain and disordered N- and C-terminal domains. The AlphaFold model is colored according to the AlphaFold confidence score (pLDDT).

Supplementary Figure S3

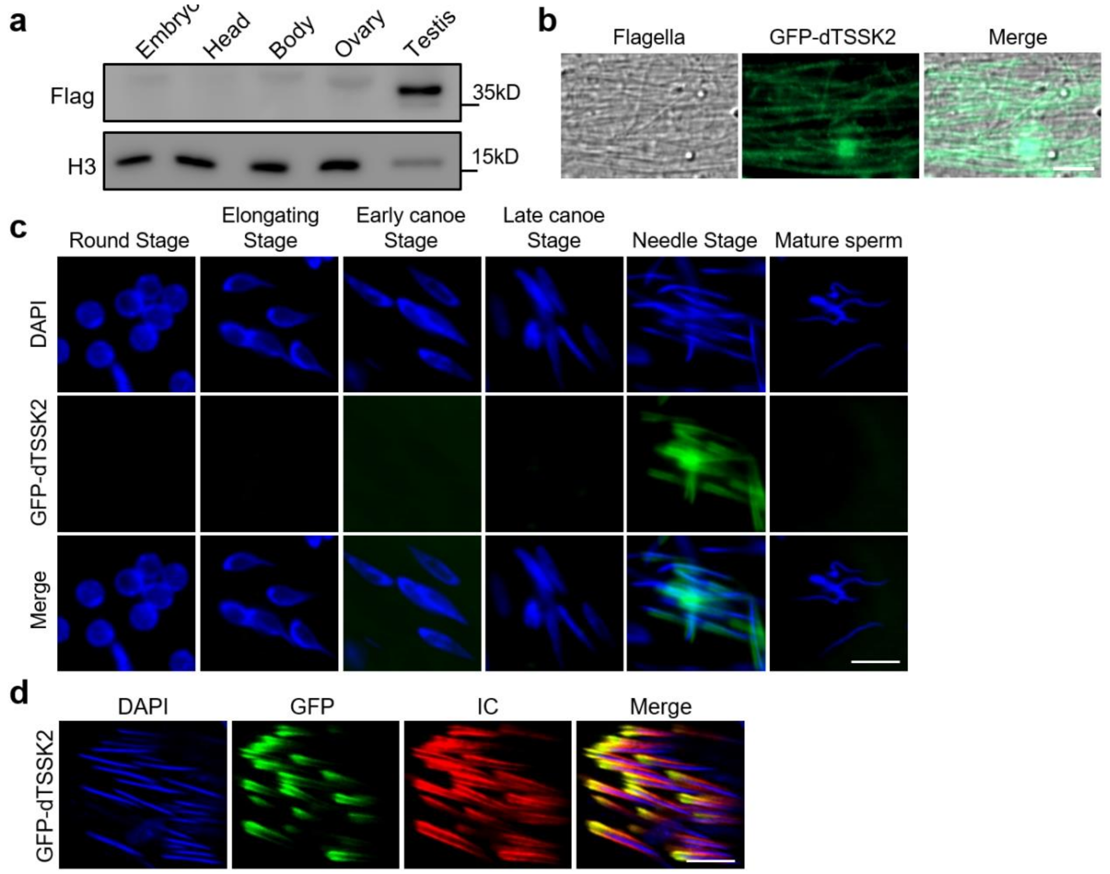

Supplementary Fig. S3. Localization of dTSSK2 during *Drosophila* spermatogenesis **a** WB analysis showing the specific expression of the dTSSK2 protein in testicular tissue. Different tissues (including embryo, head, body, ovary, and testis) were dissected from dTSSK2 transgenetic flies tagged with Flag and driven by its endogenous promoter. Tissue homogenates were used for WB analysis against anti-Flag, and the observed band size of Flag-dTSSK is consistent with its predicted size. H3 was used as a loading control. **b** Live imaging showing the distribution of dTSSK2 protein in flagellar of GFP-dTSSK2 transgenic flies (GFP-dTSSK2, green). Scale bar, 10 μm. **c** Live imaging showing the distribution of dTSSK protein of spermiogenesis from the round state to the needle stage (sperm DNA stained with DAPI, blue; GFP-dTSSK2, green). Scale bar, 10 μm. **d** Live imaging showing the distribution of dTSSK2protein of IC phase during spermiogenesis (sperm DNA stained with DAPI, blue; GFP-dTSSK2, green; ICs stained with phalloidin, red). Scale bar, 10 μm.

Supplementary Figure S4

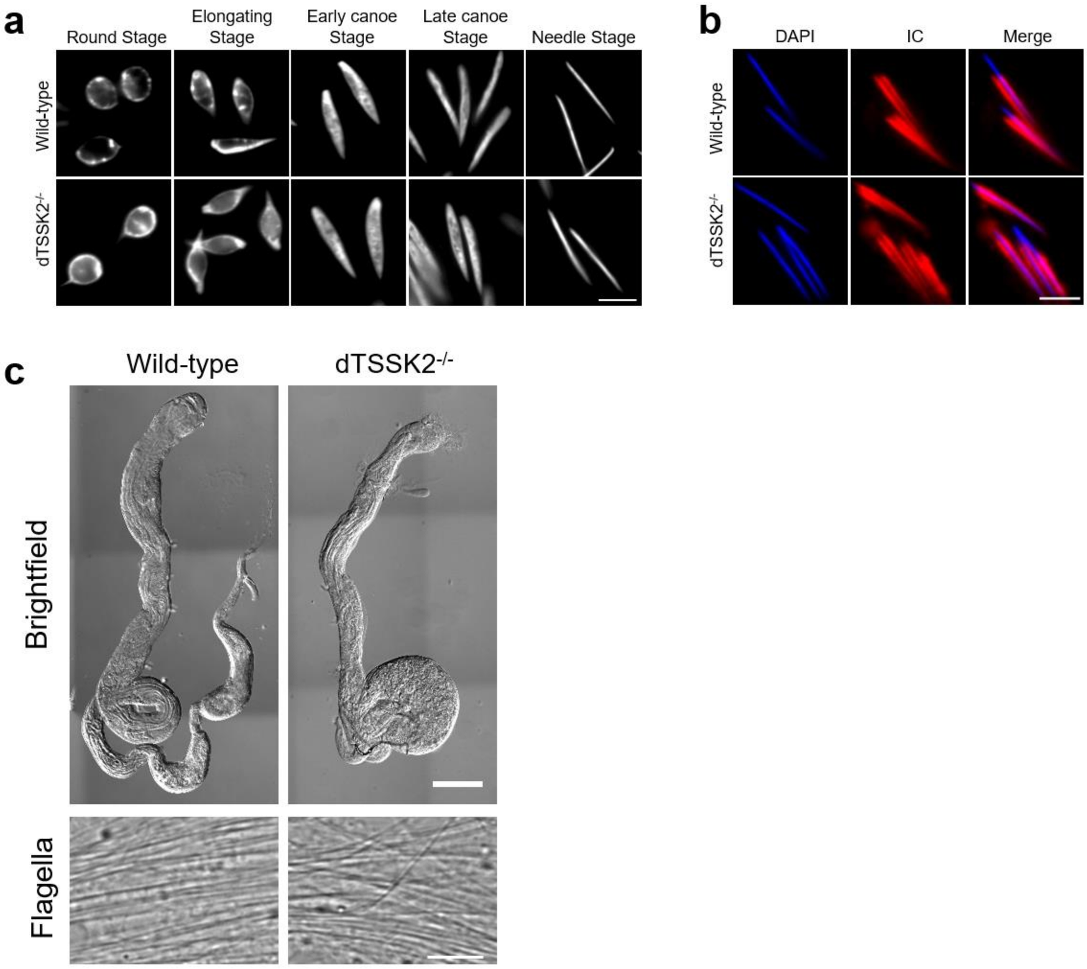

Supplementary Fig. S4. Effects of dTSSK2 deletion on spermatogenic processes

a Sperm nuclear morphology at different stages of spermiogenesis in wild-type (w^{1118}) and dTSSK2$^{-/-}$ flies. Scale bar, 10 µm. **b** Phalloidin staining of spermatids of wild-type (w^{1118}) and dTSSK2$^{-/-}$ flies. ICs stained with phalloidin, red. Nuclei stained with DAPI, blue. Scale bar, 10 µm. **c** ytological examination showing defects of flagellar arrangement in wild-type (w^{1118}) and dTSSK2$^{-/-}$ flies. The upper panel shows the morphology of the whole testis in wild-type (w^{1118}) and dTSSK2$^{-/-}$ flies. Scale bar, 100 µm. The lower panel shows the arrangement of a small patch of flagella in testicular tissue. Scale bar, 10 µm.

Supplementary Figure S5

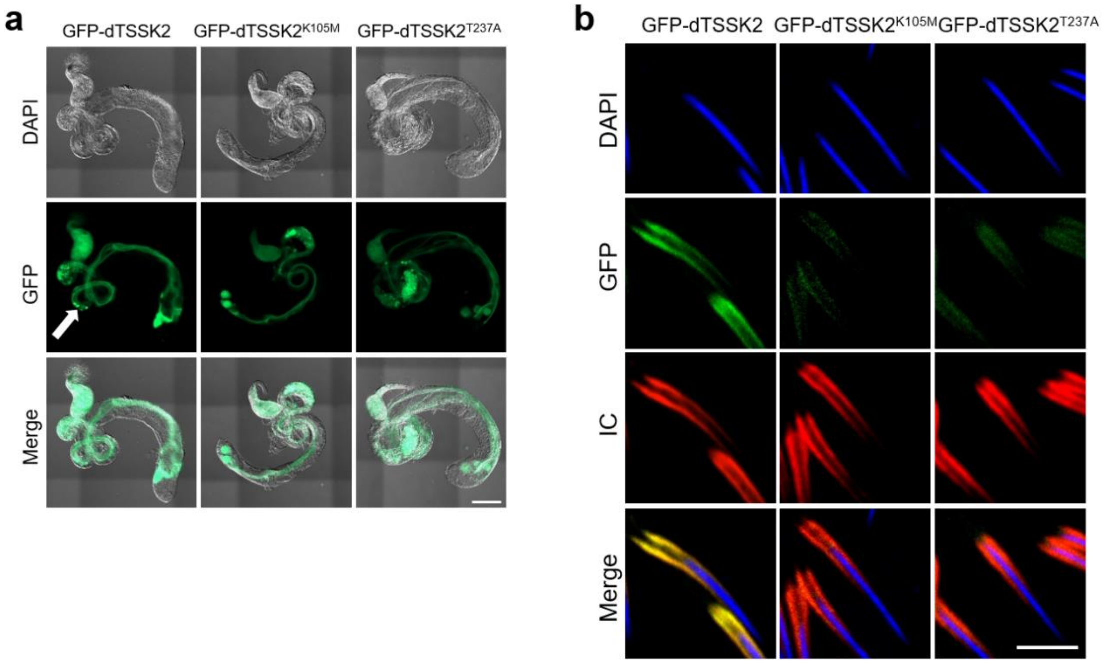

Supplementary Fig. S5. Kinase catalytic activity of dTSSK2 is important for its expression

a Live imaging showing the distribution of dTSSK2 protein in testes of GFP-dTSSK2, Flag-dTSSK2^{K105M} and Flag-dTSSK2^{T237A} transgenic flies (GFP-dTSSK2, green). Sperm bundles are indicated by the white arrow. Scale bar, 100 μm. **b** Live imaging showing the distribution of dTSSK2 protein of IC phase during spermiogenesis of GFP-dTSSK2, Flag-dTSSK2^{K105M} and Flag-dTSSK2^{T237A} transgenic flies (sperm DNA stained with DAPI, blue; GFP-dTSSK2, green; ICs stained with phalloidin, red). Scale bar, 5 μm.

Supplementary Figure S6

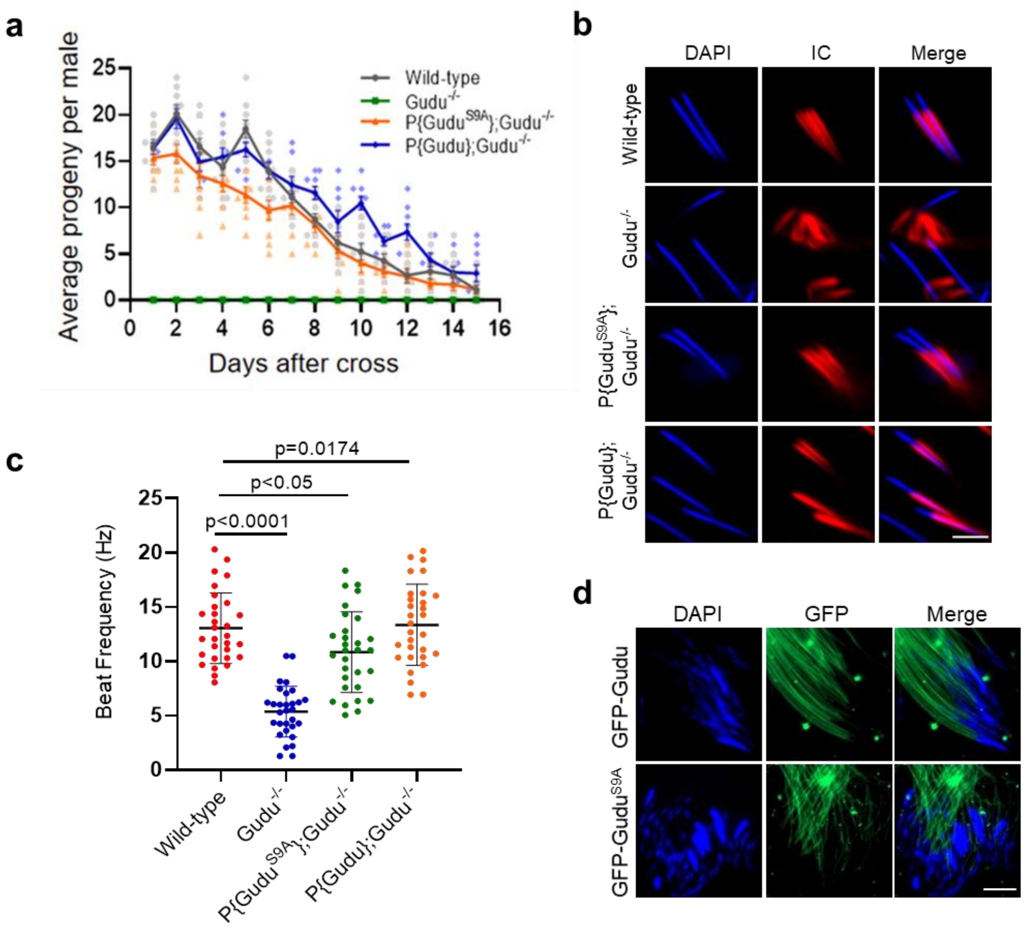

Supplementary Fig. S6. Phosphorylation of Gudu by dTSSK2 contributes to sperm motility

a Qualitative fertility assay of wild-type (w^{1118}), Gudu$^{-/-}$, P{Gudu};Gudu$^{-/-}$ and P{Gudu^{S9A}};Gudu$^{-/-}$ male flies (*n*=10 per group). Data are mean ± SEM. **b** Phalloidin staining of spermatids of dTSSK2 protein of IC phase during spermiogenesis of wild-type (w^{1118}), Gudu$^{-/-}$, P{Gudu};Gudu$^{-/-}$ and P{Gudu^{S9A}};Gudu$^{-/-}$ male flies (sperm DNA stained with DAPI, blue; ICs stained with phalloidin, red). Scale bar, 5 µm. **c** Quantification of tail-beat frequency of sperm by wild-type (w^{1118}), Gudu$^{-/-}$, P{Gudu^{S9A}};Gudu$^{-/-}$ and P{Gudu};Gudu$^{-/-}$ male flies (n = 30 per group). Data are mean ± SEM. **d** Live imaging showing the localization of Gudu protein of P{GFP-Gudu} and P{GFP-Gudu^{S9A}} male flies. sperm DNA stained with DAPI, blue; GFP, green. Scale bar, 10 µm.

Supplementary Figure S7

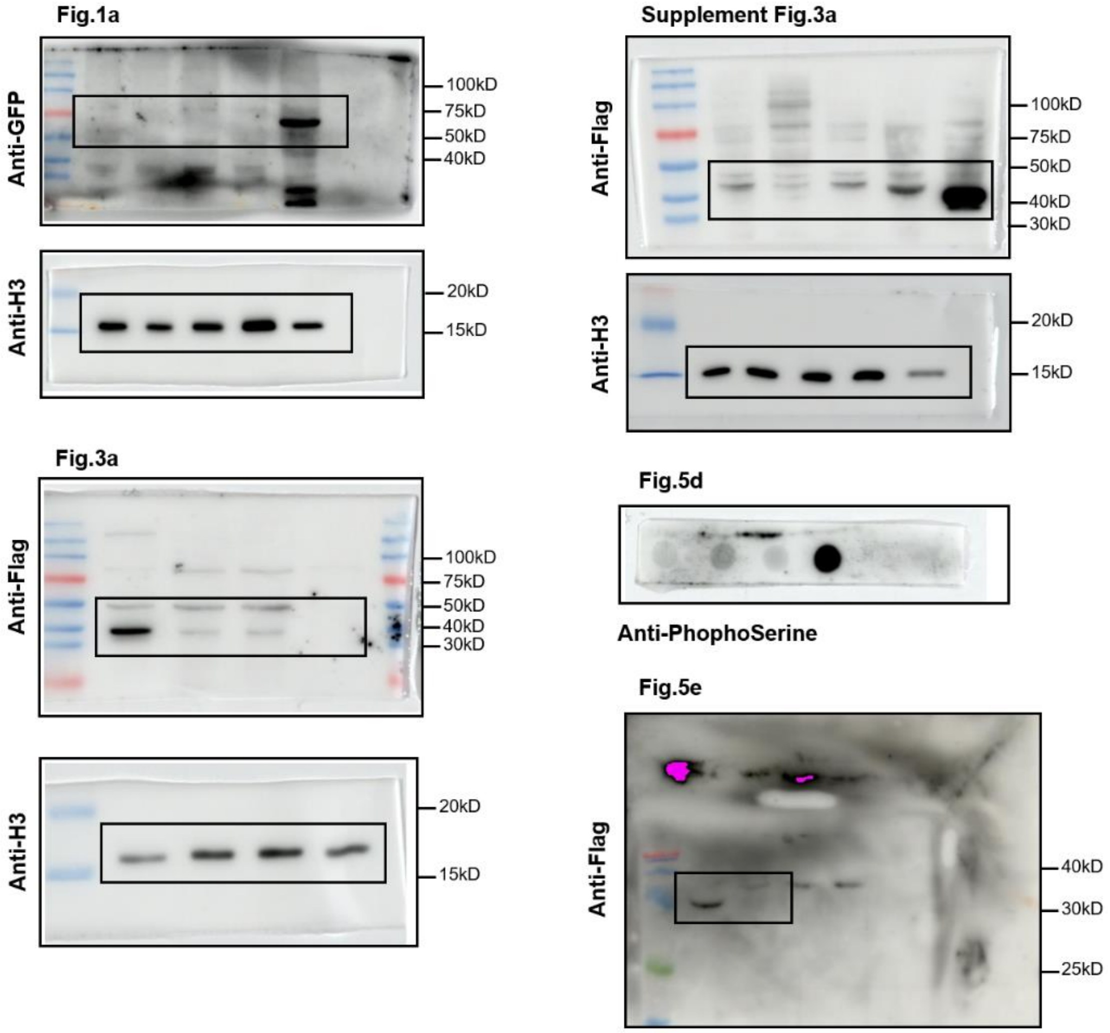

Supplementary Fig. S7 Unprocessed original images of immunoblots and gels.

Supplementary Table 1. Potential molecular substrates of CG9222 phosphorylation in phosphoproteomics results

The table shows the putative major phosphorylation substrates and corresponding sites of dTSSK2 based on phosphoproteomics results.

Gene name	Phosphorylation sites	Gene name	Phosphorylation sites
CCY	S685, S686	*CG15824*	S1856
CG10589	S329	*CG4415*	S44
CG13131	S62	*CG34021*	S141
CG13843	S341, S344	*hole*	S51
CG10793	S196	*Acn*	S36
CG3062	S235	*gudu*	S6, S9
CG31025	S543	*lark*	S300
CG31858	S201	*larp*	S218
CG32119	S474	*nsl1*	S21
CG3950	S1053	*NTPase*	S19, S20
CG4836	S242	*ORY*	S908
CG6262	S167, S435	*PifiB*	S252, S351
CG6614	S170	*snama*	S769, S772
CG6943	S280	*mod*	S330
CG3213	S286, S459	*sowah*	S423
CG7208	S181	*Kl-3*	S17, S29, S30
CG7856	S621	*Kl-5*	S2796, S2800
CG9095	S36	*LamC*	S17
Cp110	S225	*Ank2*	S1552, S1554
CRMP	S2	*Spn*	S218
ctrip	S258	*pkd2*	S123
Dek	S49, S92	*vrs*	S267
Duba	S183	*CG12289*	S318
exu	S436	*nis*	S199
fan	S169	*Wdr62*	S660
garz	S578	*Patronin*	S1192, S1194

Supplementary Table 2. The significantly down-regulated phosphorylation sites in dTSSK2$^{-/-}$ (Pvalue < 0.05, log2FC < -0.58; Statistical analyses were performed by two-sided Student's t-test.)

RowNames	logFC	P.Value
Akap200_69	-9.023954529	0.046703284
Ank2_3194	-8.232928167	0.056384234
anon-WO0118547.344_287	-1.77344755	0.015424487
anon-WO0140519.135_206	-3.355777501	0.004574851
BcDNA:AT27976_140	-10.60687387	0.006928348
BcDNA:AT27976_141	-12.29449418	0.009707773
BcDNA:AT27976_150	-11.72889188	0.014380455
CCY_1326	-2.028534829	0.030345892
CCY_603	-7.213321407	0.05025247
CG10589_329	-6.684309728	0.046721456
CG11369_312	-8.582390019	0.048346503
CG11663_126	-2.452954392	0.02258764
CG13010_100	-2.819492258	0.003603525
CG13010_104	-2.993135857	0.007448959
CG13010_130	-3.051063484	0.013877431
CG13010_133	-3.19400206	0.013522862
CG17093_196	-5.109285143	0.009971499
CG17093_324	-9.664515425	0.004243759
CG17118_246	-10.00178855	0.048200707
CG17717_2	-2.923151019	0.004165336
CG2100_41	-2.261472302	0.018188071
CG30460_1500	-1.864065482	0.050375746
CG30460_1501	-1.864065482	0.050375746
CG31245_83	-2.202030833	0.020076443
CG31858_201	-7.698330676	0.048414453
CG3517_374	-1.61850101	0.042656774
CG4415_44	-9.358572667	0.055622925
CG4836_242	-4.610260688	0.040189444
CG5103_104	-9.063289942	0.056974858
CG6409_25	-9.331855956	0.043791495
CG6614_170	-9.798964579	0.031832926
CG7208_181	-9.090455667	0.029504074
CG7326_478	-8.812082118	0.031999347
CG7856_621	-9.466263693	0.024311789
CG8136_7	-2.194166155	0.027295131
CG8509_364	-2.031520557	0.024277374
CG8509_368	-3.377483783	0.037124239
CG9216_63	-7.621276585	0.055736451
CG9222_19	-11.39331553	0.001194836
CG9222_20	-12.06833202	0.000963715

CG9222_237	-2.962490697	0.008984446
CG9222_282	-3.80089146	0.001695741
Ciz1_851	-1.988918581	0.02001869
Ciz1_855	-1.926773534	0.023599022
cmb_1584	-2.387264292	0.016889088
Cp110_225	-8.367575534	0.045238205
Dek_49	-10.24887763	0.003566484
Dek_92	-7.715017592	0.032997955
Dek_94	-1.904778452	0.015611934
Dhc98D_355	-1.946869026	0.022982771
Glys_671	-2.147150136	0.051249137
gudu_6	-8.998566809	0.020333808
gudu_9	-3.359160684	0.007706411
KrT95D_321	-7.456364552	0.055144335
mod_330	-3.390134057	0.001298349
ms(2)34Fe_684	-1.315479866	0.050519676
nis_196	-2.506712799	0.039040559
nis_199	-2.756197295	0.023288493
pcm_1517	-9.11797923	0.051418766
Reg-5_60	-9.163652355	0.045380506
Rsbp15_271	-1.538989132	0.058362588
snama_769	-9.377268294	0.019260329
snama_772	-9.377268294	0.019260329
Sos-RA_1383	-8.684009288	0.045909216
ZnT35C_216	-8.528727125	0.037445774

The expression level of proteins with down-regulated major phosphorylation modification levels was no significant change in dTSSK2$^{-/-}$ (Pvalue > 0.05; Statistical analyses were performed by two-sided Student's t-test.).

name	ID	CG9222_vs_w118_p.val	CG9222_vs_w118_significant
Ank2	Q24241	0.081408126	FALSE
CCY	A0A0S0X8W1	0.30786128	FALSE
CG10589	Q9VP96	0.828551445	FALSE
CG13131	Q8T8X9	0.450830301	FALSE
CG13843	Q9VCX6	0.953641388	FALSE
CG15824	Q9VPV1	0.80222823	FALSE
CG3062	Q9W4I5	0.350914388	FALSE
CG31025	Q6NN96	0.988997807	FALSE
CG34021	A1Z8X1	0.45530122	FALSE
CG3950	M9PDW8	0.403783049	FALSE
CG4415	Q8T4F0	0.855263898	FALSE
CG4836-RD	E0R969	0.961068265	FALSE
CG6262	Q7YTX8	0.251382699	FALSE
CG6614	X2J5P7	0.000241903	FALSE

CRMP	Q8IPQ2	0.270022405	FALSE
ctrip	F6M9W1	0.414735313	FALSE
exu	E1JGN9	0.269689046	FALSE
fan	Q9VSD3	0.309707224	FALSE
garz	A1Z8W8	0.426068766	FALSE
gudu	Q9VM21	0.063000906	FALSE
kl-3	A8Y5B7	0.062976468	FALSE
kl-5	Q5LJN5	0.060180429	FALSE
LamC	Q03427	0.949097302	FALSE
lark	Q94901	0.552943327	FALSE
larp	A0A0B4K7Y7	0.200296147	FALSE
mod	P13469	0.63602676	FALSE
nis	Q9VQ22	0.675343988	FALSE
NTPase	O76268	0.201807648	FALSE
ORY	Q5LJN3	0.089229137	FALSE
Pkd2	Q9VK95	0.407097419	FALSE
vrs	Q9VG65	0.024525649	FALSE

Supplementary Table 3. Flies used in this study

Organisms/strains	SOURCE	IDENTIFIER
D. melanogaster: w^{1118}	BDSC	3605
D. melanogaster: *attP40*	BDSC	25709
D. melanogaster: *attP2*	BDSC	25710
D. melanogaster: w; P{Flag-GFP-dTSSK2}	This paper	N/A
D. melanogaster: w; dTSSK2$^{-/-}$	This paper	N/A
D. melanogaster: w; P{dTSSK2};dTSSK2$^{-/-}$	This paper	N/A
D. melanogaster: w; P{Mst35Bb-GFP}	This paper	N/A
D. melanogaster: w; P{Mst35Bb-GFP}; dTSSK2$^{-/-}$	This paper	N/A
D. melanogaster: w; P{dTSSK2^{K105M}}	This paper	N/A
D. melanogaster: w; P{dTSSK2^{K105M}}; dTSSK2$^{-/-}$	This paper	N/A
D. melanogaster: w; P{dTSSK2^{T237A}}	This paper	N/A
D. melanogaster: w; P{dTSSK2^{T237A}}; dTSSK2$^{-/-}$	This paper	N/A
D. melanogaster: w; P{Flag-GFP-dTSSK2^{K105M}}	This paper	N/A
D. melanogaster: w; P{Flag-GFP-dTSSK2^{K105M}}; dTSSK2$^{-/-}$	This paper	N/A
D. melanogaster: w; P{Flag-GFP-dTSSK2^{T237A}}	This paper	N/A
D. melanogaster: w; P{Flag-GFP-dTSSK2^{T237A}}; dTSSK2$^{-/-}$	This paper	N/A
D. melanogaster: w; P{TSSK4}	This paper	N/A
D. melanogaster: w; P{TSSK4}; dTSSK2$^{-/-}$	This paper	N/A
D. melanogaster: w; P{Flag-GFP-Gudu}	This paper	N/A
D. melanogaster: w; P{Flag-GFP-Gudu^{S9A}}	This paper	N/A
D. melanogaster: w; Gudu$^{-/-}$	This paper	N/A
D. melanogaster: w; P{Gudu}	This paper	N/A
D. melanogaster: w; P{Gudu^{S9A}}	This paper	N/A
D. melanogaster: w; P{Gudu}; Gudu$^{-/-}$	This paper	N/A
D. melanogaster: w; P{Gudu^{S9A}}; Gudu$^{-/-}$	This paper	N/A
D. melanogaster: w; P{Flag-Gudu}	This paper	N/A
D. melanogaster: w; P{Flag-Gudu}; dTSSK2$^{-/-}$	This paper	N/A

Supplementary Table 4. Oligonucleotides used in this study

**Primers used for transgene**	
CG9222-pro-F	5'-TAGACGGCGGCCGCACTTGAACTCCACGCCGTATTCGTC-3'
CG9222-pro-R	5'-TAGACGCTCGAGTAAAAAGGGTACCACAAGTACGGTT-3'
CG9222-F	5'-TAGACGACTAGTATGGCGACATCTCCCAGAAGAAAAA-3'
CG9222-R	5'-TAGACGGGCGCGCCAATCAGAAACCGAGTCAACGTGAGA-3'
GFP-F	5'-ACCGGTATGGACTACAAAGACGACGATGACA-3'
GFP-R	5'-ACTAGTCTTGTACAGCTCGTCCATGCC-3'
Mst35Bb-F	5'-GAATTCGTCTACTCTCCCTGTGTGCGTGC-3'
Mst35Bb-R	5'-CTCGAGCTTGCAAATCCGTCGGCGCT-3'
Mst35Bb-3'UTR-F	5'-GCGGCCGCTACTGAGATAGGAAAAAGTCTGATGCAGTT-3'
Mst35Bb-3'UTR-R	5'-GGTACCCGAAAGAAAGAACTAAGAGTTTCTCTTCACAT-3'
Gudu-pro-F	5'-GAATTCGTTAACAGATCTGCGGCCGCGCTTGCCACTCGCATTTC-3'
Gudu-pro-R	5'-CGTCGTCTTTGTAGTCCATACCGGTCCTTTCGCGTTGGATTACT-3'
Gudu-F	5'-CATGGACGAGCTGTACAAGCTCGAGATGATTGGCACTAGCAGCG-3'
Gudu-R	5'-GGGTGCGAAGGGAAACCGGGGTACCGGATGAGCATTTCCACCAG-3'
dTSSK2^{K105M}-F	5'-GAAAGCGGGTGGCTGTCATGATCATATCCAAAGTGAAGGCTCCC-3'
dTSSK2^{K105M}-R	5'-GACAGCCACCCGCTTTCCGTACTCC-3'
dTSSK2^{T237A}-F	5'-TACTCTCGAAAGCCTTCTGCGGCAGCTATG-3'
dTSSK2^{T237A}-R	5'-GCAGAAGGCTTTCGAGAGTATCACTTGGTTGTCG-3'
Gudu^{S9A}-F	5'-GCAGCGGAACGGCCCACAATCGGAGT-3'
Gudu^{S9A}-R	5'-CCGATTGTGGGCCGTTCCGCTGCTA-3'
**Primers used for fly mutants**	
CG9222-gRNA1-F	5'-TTCgGAAAAAACGATCAAGGCGG-3'
CG9222-gRNA1-R	5'-AAACCCGCCTTGATCGTTTTTTCC-3'
CG9222-gRNA2-F	5'-AAACTCTGCTCCTGACGACGACCC-3'
CG9222-gRNA2-R	5'-AAACTCTGCTCCTGACGACGACCC-3'
Gudu-gRNA-F	5'-GAAAGATATCCGGGTGAACTTCGGAGCGGAACGTCCCACAATGTTTTAGAGCTAGAAATAGCAAGTTAAAATAAGG-3'
Gudu-gRNA-R	5'-CTATTTCTAGCTCTAAAACCCGTGGTATCCGAGTCCTCCGACGTTAAATTGAAAATAGGTCTATATATACG-3'
