# Supplementary material for: Testis-specific serine/threonine kinase dTSSK2 regulates sperm motility and male fertility in Drosophila
Source: Commun Biol. 2025 May 8;8:710. doi: 10.1038/s42003-025-08163-z (PMC12059139; doi:10.1038/s42003-025-08163-z)
Supplement: Supplementary file 2 — Description of Additional Supplementary Files [file 42003_2025_8163_MOESM2_ESM.docx]

**Description of Additional Supplementary Files**

File name: Supplementary Data 1

Description: The source data behind the graphs in the paper
