# Supplementary material for: Testis-specific serine/threonine kinase dTSSK2 regulates sperm motility and male fertility in Drosophila
Source: Commun Biol. 2025 May 8;8:710. doi: 10.1038/s42003-025-08163-z (PMC12059139; doi:10.1038/s42003-025-08163-z)
Supplement: Supplementary file 4 — Reporting Summary [file 42003_2025_8163_MOESM4_ESM.pdf]

nature portfolio

Corresponding author(s): Gao Guanjun

Last updated by author(s): Apr 29, 2025

# Reporting Summary

Nature Portfolio wishes to improve the reproducibility of the work that we publish. This form provides structure for consistency and transparency in reporting. For further information on Nature Portfolio policies, see our Editorial Policies and the Editorial Policy Checklist.

## Statistics

For all statistical analyses, confirm that the following items are present in the figure legend, table legend, main text, or Methods section.

| n/a | Confirmed | |
|---|---|---|
| ☐ | ☒ | The exact sample size (*n*) for each experimental group/condition, given as a discrete number and unit of measurement |
| ☐ | ☒ | A statement on whether measurements were taken from distinct samples or whether the same sample was measured repeatedly |
| ☐ | ☒ | The statistical test(s) used AND whether they are one- or two-sided<br>*Only common tests should be described solely by name; describe more complex techniques in the Methods section.* |
| ☐ | ☒ | A description of all covariates tested |
| ☐ | ☒ | A description of any assumptions or corrections, such as tests of normality and adjustment for multiple comparisons |
| ☐ | ☒ | A full description of the statistical parameters including central tendency (e.g. means) or other basic estimates (e.g. regression coefficient) AND variation (e.g. standard deviation) or associated estimates of uncertainty (e.g. confidence intervals) |
| ☐ | ☒ | For null hypothesis testing, the test statistic (e.g. *F*, *t*, *r*) with confidence intervals, effect sizes, degrees of freedom and *P* value noted<br>*Give P values as exact values whenever suitable.* |
| ☒ | ☐ | For Bayesian analysis, information on the choice of priors and Markov chain Monte Carlo settings |
| ☒ | ☐ | For hierarchical and complex designs, identification of the appropriate level for tests and full reporting of outcomes |
| ☒ | ☐ | Estimates of effect sizes (e.g. Cohen's *d*, Pearson's *r*), indicating how they were calculated |

*Our web collection on statistics for biologists contains articles on many of the points above.*

## Software and code

Policy information about availability of computer code

| | |
|---|---|
| Data collection | Proteomic analysis using an Easy-nLC 1000 system coupled with a Q Exactive HF (Thermo Scientific) mass spectrometer. Peptide separation and analysis were performed using an Easy-nLC 1200 system coupled with an Orbitrap Fusion mass spectrometer (Thermo Scientific). |
| Data analysis | Immunofluorescence imaging was analyzed using ZEISS ZEN 3.7 and Fiji software. Sequence comparison and phylogenetic tree analyses were performed using MEGA_11.0.13 and GeneDoc. Statistical graph analyses were performed using Prism v8.0.2.263. Statistical analyses were perfored using R version 4.2.3. |

For manuscripts utilizing custom algorithms or software that are central to the research but not yet described in published literature, software must be made available to editors and reviewers. We strongly encourage code deposition in a community repository (e.g. GitHub). See the Nature Portfolio guidelines for submitting code & software for further information.

## Data

Policy information about availability of data

All manuscripts must include a data availability statement. This statement should provide the following information, where applicable:

- Accession codes, unique identifiers, or web links for publicly available datasets
- A description of any restrictions on data availability
- For clinical datasets or third party data, please ensure that the statement adheres to our policy

Mass spectrometry proteomics data have been deposited to the iProX storage platform (http://www.iprox.cn/page/HMV006.html/) with the dataset identifier PX0010619000.

## Research involving human participants, their data, or biological material

Policy information about studies with human participants or human data. See also policy information about sex, gender (identity/presentation), and sexual orientation and race, ethnicity and racism.

| | |
|---|---|
| Reporting on sex and gender | NA |
| Reporting on race, ethnicity, or other socially relevant groupings | NA |
| Population characteristics | NA |
| Recruitment | NA |
| Ethics oversight | NA |

Note that full information on the approval of the study protocol must also be provided in the manuscript.

# Field-specific reporting

Please select the one below that is the best fit for your research. If you are not sure, read the appropriate sections before making your selection.

☒ Life sciences ☐ Behavioural & social sciences ☐ Ecological, evolutionary & environmental sciences

For a reference copy of the document with all sections, see nature.com/documents/nr-reporting-summary-flat.pdf

# Life sciences study design

All studies must disclose on these points even when the disclosure is negative.

| | |
|---|---|
| Sample size | We prespecified the sample size through experimental analysis |
| Data exclusions | No data were excluded from the data set |
| Replication | The data were repeated on multiple independent animals. All replicates are true biological replicates |
| Randomization | Animals were randomly allocated to control or treatment groups |
| Blinding | In most cases, detailed in the methods sections, samples were analysed by a blinded observer, |

# Reporting for specific materials, systems and methods

We require information from authors about some types of materials, experimental systems and methods used in many studies. Here, indicate whether each material, system or method listed is relevant to your study. If you are not sure if a list item applies to your research, read the appropriate section before selecting a response.

## Materials & experimental systems

| n/a | Involved in the study |
| --- | --- |
| ☐ | ☒ Antibodies |
| ☒ | ☐ Eukaryotic cell lines |
| ☒ | ☐ Palaeontology and archaeology |
| ☐ | ☒ Animals and other organisms |
| ☒ | ☐ Clinical data |
| ☒ | ☐ Dual use research of concern |
| ☒ | ☐ Plants |

## Methods

| n/a | Involved in the study |
| --- | --- |
| ☒ | ☐ ChIP-seq |
| ☒ | ☐ Flow cytometry |
| ☒ | ☐ MRI-based neuroimaging |

# Antibodies

| | |
| --- | --- |
| Antibodies used | Anti-H3 antibody Abcam Cat# ab1791<br>Anti-GFP antibody Abcam Cat# ab290<br>Anti-Flag antibody Sigma Cat# H7435<br>Anti-Phosphoserine Merck Cat# 05-1000<br>Peroxidase AffiniPure Goat Anti-Mouse IgG(H+L) Yeasen Cat# 33201ES60<br>Peroxidase-Conjugated Goat Anti-Rabbit IgG (H+L) Yeasen Cat# 33101ES60 |
| Validation | Each primary antibody used in this paper was validated by the manufacturing company and laboratory before it could be used |

# Animals and other research organisms

Policy information about studies involving animals; ARRIVE guidelines recommended for reporting animal research, and Sex and Gender in Research

| | |
| --- | --- |
| Laboratory animals | In this thesis, Drosophila melanogaster was mainly used as the experimental model. Different transgenic and mutant flies were obtained in the laboratory |
| Wild animals | No wild-type experimental animals exist in this paper |
| Reporting on sex | This paper is not related to gender content |
| Field-collected samples | The laboratory environment and requirements were in accordance with the requirements of a standard research institution |
| Ethics oversight | No ethical approval or guidance was required Because the whole content of this paper is about the fertility of Drosophila melanogaster |

Note that full information on the approval of the study protocol must also be provided in the manuscript.

# Plants

| | |
| --- | --- |
| Seed stocks | *Report on the source of all seed stocks or other plant material used. If applicable, state the seed stock centre and catalogue number. If plant specimens were collected from the field, describe the collection location, date and sampling procedures.* |
| Novel plant genotypes | *Describe the methods by which all novel plant genotypes were produced. This includes those generated by transgenic approaches, gene editing, chemical/radiation-based mutagenesis and hybridization. For transgenic lines, describe the transformation method, the number of independent lines analyzed and the generation upon which experiments were performed. For gene-edited lines, describe the editor used, the endogenous sequence targeted for editing, the targeting guide RNA sequence (if applicable) and how the editor was applied.* |
| Authentication | *Describe any authentication procedures for each seed stock used or novel genotype generated. Describe any experiments used to assess the effect of a mutation and, where applicable, how potential secondary effects (e.g. second site T-DNA insertions, mosiacism, off-target gene editing) were examined.* |
